# Melancholia. Historical evolution through a case report

**DOI:** 10.1192/j.eurpsy.2021.2028

**Published:** 2021-08-13

**Authors:** P. Coucheiro Limeres, L. Amaya Lega, A. Franco Soler, A. Cerame Del Campo

**Affiliations:** Psychiatry, Instituto Psiquiatrico José Germain, Leganés, Spain

**Keywords:** bipolar disorder, Historical evolution, Melancholia, psychotic depression

## Abstract

**Introduction:**

The diagnosis of psychotic depression has its origin in the millennial term of Melancholia.

**Objectives:**

A case of psychotic depression is presented to highlight its psychopathological characteristics and to make a historical overview of its origins.

**Methods:**

We present the case of a 40-year-old male patient with a history of dysthymic mood who developed a major depressive mood, loss of self-care, decreased apetite, insomnia and repetitive speech with ideas of guilt and ruin of psychotic characteristics.

**Results:**

Melancholy is a term used since the time of Hippocrates, who spoke of it as the state that appears after the prolongation of an intense period of sadness. It was extolled and self-attributed by authors such as Montaigne and branded as selfish by authors such as Cicero in the days when reason and madness formed a whole and distinguishing their limits was a complex task. Esquirol changed his name to Lypemania to get rid of its poetic nuances and framed it within partial insanity. Both he and the rest of the psychopathologists of the XIX century and early XX considered the melancholic as the great tormented, the one who despises himself and blames all ills, who suffers from apathy and above all presents a strong pain of the soul.

**Conclusions:**

Later it was Falret and Baillarger who unified melancholy with mania in what they nominate as circular and dual-form insanity. This gave way to the Krapelinian entity of manic-depressive insanity, the direct predecessor of the current Bipolar Disorder, which includes the diagnosis of our clinical case.

**Disclosure:**

No significant relationships.

